# Can gender difference in prescription drug use be explained by gender-related morbidity?: a study on a Swedish population during 2006

**DOI:** 10.1186/1471-2458-14-329

**Published:** 2014-04-08

**Authors:** Jessica Skoog, Patrik Midlöv, Lars Borgquist, Jan Sundquist, Anders Halling

**Affiliations:** 1 Department of Clinical Sciences in Malmö, Center for Primary Health Care Research, Lund University, SE-205 02 Malmö, Sweden; 2 Department of Medical and Health Sciences, General Practice, Linköping University, SE-581 83 Linköping, Sweden; 3 Stanford Prevention Research Center, Stanford University School of Medicine, Stanford, CA, USA; 4 Research Unit for General Practice, Institute of Public Health, University of Southern Denmark, J.B. Winsløws Vej 9A, DK-5000 Odense C, Denmark

**Keywords:** Prescription drugs, Multi-morbidity, Gender difference, Gender-related morbidity

## Abstract

**Background:**

It has been reported that there is a difference in drug prescription between males and females. Even after adjustment for multi-morbidity, females tend to use more prescription drugs compared to males. In this study, we wanted to analyse whether the gender difference in drug treatment could be explained by gender-related morbidity.

**Methods:**

Data was collected on all individuals 20 years and older in the county of Östergötland in Sweden. The Johns Hopkins ACG Case-Mix System was used to calculate individual level of multi-morbidity. A report from the Swedish National Institute of Public Health using the WHO term DALY was the basis for gender-related morbidity. Prescription drugs used to treat diseases that mainly affect females were excluded from the analyses.

**Results:**

The odds of having prescription drugs for males, compared to females, increased from 0.45 (95% confidence interval (CI) 0.44-0.46) to 0.82 (95% CI 0.81-0.83) after exclusion of prescription drugs that are used to treat diseases that mainly affect females.

**Conclusion:**

Gender-related morbidity and the use of anti-conception drugs may explain a large part of the difference in prescription drug use between males and females but still there remains a difference between the genders at 18%. This implicates that it is of importance to take the gender-related morbidity into consideration, and to exclude anti-conception drugs, when performing studies regarding difference in drug use between the genders.

## Background

Prescription of drugs is one of the health care system’s most important methods to treat, relieve and sometimes cure diseases. Nevertheless, prescription drug treatment may carry risks such as adverse drug reactions, interactions and polypharmacy [1], circumstances that may lead to hospital admission [2,3]. Beyond the fact that treatment with prescription drugs may cause patients to suffer from side effects, these unwanted effects are the source of substantial expenses to society. The total cost of prescription drugs is very high. In 2012 the total drug cost for prescription drugs in Sweden amounted to SEK 36 billion (≈ 3.6 billion Euros) [4]. Thus, on behalf of both the society and patients, it is important to keep the prescription of drugs secure, effective and well motivated.

There are significant gender differences in health care utilization and drug prescription between the genders in Sweden, Europe and the United States. In general, females use more prescription drugs [5-12] and more health care than males [13-15]. In an earlier study we showed that the gender difference in drug prescription remains after adjustment for multi-morbidity. Males had less than half the odds of having prescription drugs compared to females despite adjustment for multi-morbidity [16]. This indicates that factors other than differences in multi-morbidity explain the higher prescription rates among females. Still, if females are afflicted to a higher extent than males with diseases that are treated with prescription drugs, morbidity may still explain the gender difference in drug prescription. Previous studies have shown that there is a difference between the diseases males and females are afflicted with [17,18]. However, it has not yet been studied whether this gender-related morbidity affects the prescription of drugs. The main objective of this study was to analyse whether the gender difference in drug treatment observed after adjustment for multi-morbidity can be explained by gender-related morbidity.

## Methods

### Study population

Data was collected in 2006 from the total population aged 20 years or older in the Swedish county of Östergötland, which had about 400,000 residents in 2006. Östergötland is situated 200 km southwest of Stockholm and the age demographic matches that of the rest of Sweden [19]. Data on the population’s age, gender and diagnoses in both primary and secondary care was obtained from the Care Data Warehouse in Östergötland (CDWÖ), a register containing information on both public and private care. This register has been described previously [20]. The study was approved by the research ethics committee at Linköping University (approval numbers 147/05 and 29/06).

### Independent variable

Multi-morbidity was calculated using the Johns Hopkins Adjusted Clinical Groups (ACG) Case-Mix System, a system based on the theory that multi-morbidity corresponds to a certain need for health care resources. This system is based on the patients’ diagnoses recorded during a defined period of time. The ethiology, duration, method of diagnosis, treatment and need of specialized care is considered for every one of the patients’ diagnoses. The ACG Case-Mix System has previously been described [21-24]. Individuals without need of health care according to the ACG Case-Mix system are placed in Resource Utilization Band 0 (RUB 0) and individuals with a very high degree of need for health care resources are placed in RUB 5. For example, preventive interventions correspond to RUB 1, a single chronic diagnosis could correspond to RUB 3 and a certain combination of chronic diagnoses corresponds to RUB 4 or RUB 5.

### Dependent variable

The use (dichotomous variable) of prescription drug(s) in 2006 was the dependent variable. Information concerning the use of prescription drugs at the individual level was acquired from the Swedish Prescribed Drug Register, which is maintained by the National Board of Health and Welfare. This register collects information from the National Corporation of Swedish Pharmacies (Apoteket AB). In 2006 Apoteket AB had a monopoly on sales of prescription drugs and all prescription drugs were tracked through Apoteket AB.

The Anatomical Therapeutic Chemical (ATC) classification system was elaborated by the WHO to enable internationally comparable studies on prescription drugs. Active substances are classified in different groups according to the organ or system on which they act and their therapeutic, pharmacological and chemical properties. The drugs are divided into 14 main ATC groups and these groups are subsequently divided into five levels [25].

Over-the-counter drugs were not included in this study.

### Analysis

We used a report from the Swedish National Institute of Public Health to identify diseases that tend to afflict females more frequently [17]. In the report, the disability-adjusted life year (DALY) was used to measure the burden of disease. The DALY concept was elaborated by the WHO and the World Bank to measure the burden of disease in the population, taking into consideration both mortality and disability [26-29]. The DALY is a time-based measure that combines years of life lost due to premature mortality and years spent living in states of less than full health. DALYs are the sum of life years lost due to premature mortality and years lived with disability, adjusted for severity. To put it simply, 1 DALY means one lost healthy year. In the report from the Swedish National Institute of Public Health, both major and minor gender differences were identified. To ensure that the prevalence of the disease and the difference between the genders were high enough to affect the results, we created two cut-off points. We selected diseases that had at least 7,500 DALYs in Sweden in 2006 for both genders combined and for which the difference between the genders was at least 20%, with a higher number of DALYs for females. Having identified the diseases that tend to afflict females to a higher extent, we identified the prescription drugs that are commonly used to treat these specific diseases (Table 1) according to Swedish National Guidelines [30,31]. Anti-conception that is commonly used by healthy females as contraceptives is a special case. These prescription drugs are not considered to treat any disease but they may explain some of the difference in prescription drug treatment between the genders. Hence, these drugs were also identified as drugs causing a difference in prescription drug treatment between the genders.

**Table 1:** ATC codes for the prescription drugs that are used to treat diseases and conditions that afflict females to a greater extent than males

**Disease or condition**	**ATC code**	**Prescription drug(s)**
Anti-conception	G03A	Anti-conception drugs
Climacteric complaints (HRT)	G03C	Estrogens
	G03D	Gestagens
Thyroid gland disorders	H03AA01	Thyroid hormones
Cystitis	J01CA08	Pivmecillinam
	J01EA01	Trimethoprim
	J01XE01	Nitrofurantoin
Osteoarthritis	M01	Anti-inflammatory and anti-rheumatic drugs
	N02BE01	Paracetamol
Migraine	N02C	Migraine drugs, including triptans
Depression and anxiety disorders	N05BA	Benzodiazepines
	N05BB01	Hydroxyzine
	N05BE	Buspirone
	N06A	Antidepressant drugs, including SSRIs
Insomnia	N05CD	Derivatives of benzodiazepines (e.g. nitrazepam)
	N05CF	Benzodiazepine-related drugs (e.g. zolpidem)
	N05CM06	Propiomazine
Asthma and COPD	R03AC	Selective beta-2 stimulants and inhalable corticosteroids
	R03AK	
	R03BA02	
	R03BA05	
	R03BB01	

### Statistics

The prescription drugs used to treat the diseases that afflict females to a higher extent, and anti-conception drugs, were excluded from the analysis using logistic regression. First, the prescription drugs were excluded one by one in subsequent univariate analyses. In the next step, the prescription drugs that caused a decrease in the gender difference, i.e. the prescription drugs that gave an odds ratio (OR) >0.45 [16] when excluded in the univariate analyses, were excluded. The OR represents the odds of having prescription drugs for males compared to females.

We used STATA version 12 (Stata Corporation, Texas, USA) for statistical analyses. Logistic regression was used to examine the odds of having prescription drugs in the study population, giving odds ratios (ORs and 95% confidence intervals (CIs). We generated three dichotomous models: Model 1 was adjusted for age and multi-morbidity; Model 2 was adjusted for age, multi-morbidity and relative ATC codes; and Model 3 was adjusted for age, multi-morbidity and ATC codes that gave an OR >0.45 when excluded in the univariate analyses.

Logistic regression gives us the odds ratio of having prescription drugs. For some diseases, it is highly likely to receive more than one prescription drug. For example in dementia, which afflicts females to a higher extent than males, there is a strong likelihood that the patient is using prescription drugs in addition to the prescription drug used to treat the dementia. The prescription drugs used to treat these diseases, for example dementia, were not excluded in this study.

## Results

This study comprised 313,977 individuals at least 20 years old. 66% of the population used at least one prescription drug. Males used prescription drugs to a lesser degree compared to females (Table 2).

**Table 2:** Characteristics of the population’s prescription drug use

		**Prescription drug use in total population**	
**Variables**		**Yes**	**No**
		**N (%)**	**N (%)**
All		205827 (66)	108150 (35)
Gender	Female	121682 (77)	37021 (23)
	Male	84145 (54)	71129 (46)
Age	20-29	23916 (51)	23289 (49)
	30-39	27666 (53)	24568 (47)
	40-49	30419 (56)	24293 (44)
	50-59	34946 (65)	19045 (35)
	60-69	36745 (73)	11376 (34)
	70-79	27643 (87)	4038 (13)
	80-	24492 (94)	1541 (6)
Multi-morbidity level	0	26822 (26)	75013 (74)
	1	30364 (69)	13491 (31)
	2	51674 (80)	12913 (20)
	3	82988 (93)	6595 (7)
	4	10775 (99)	126 (1)
	5	3204 (99.6)	12 (0.4)

N – Number of observations.

The greatest gender differences in morbidity with a preponderance for females were for the diagnoses Depression and Anxiety disorders, Osteoarthritis, Migraine, Insomnia, Asthma and COPD, Thyroid gland disorders and Cystitis.

The gender difference in having prescription drugs was greatest at young age and decreased with age (Table 3).

**Table 3:** Odds ratios of having prescription drugs for males in different age categories after adjustment for multi-morbidity

**Age (years)**	**OR (95% CI)**	**P-value**
20-39	0.32 (0.31-0.33)	<0.001
40-59	0.52 (0.50-0.54)	<0.001
60-79	0.60 (0.57-0.62)	<0.001
80-	0.62 (0.55-0.69)	<0.001

After adjustment for multi-morbidity, males had less than half the odds of using prescription drugs compared to females (OR 0.45 (95% CI 0.45-0.46)). After excluding anti-conception drugs, the odds ratio increased (OR 0.65 (95% CI 0.64-0.66)). The effect on the odds ratios for other prescription drugs is shown in Table 4. After excluding all the prescription drugs that gave an odds ratio >0.45 when excluded in the univariate analyses (“List of prescription drugs excluded in Model 3”), the total OR of having prescription drugs for males, compared to females, increased further to 0.82 (95% CI 0.80-0.83).

**Table 4:** Odds ratios of having prescription drugs for males, compared to females, after adjustment for age, multi-morbidity and relevant prescription drug in univariate analyses (Model 2)

**Prescription drug excluded from the analysis**	**OR (95% CI)**	**P-value**
NSAIDs	0.44 (0.43-0.45)	<0.001
Buspirone	0.45 (0.45-0.46)	<0.001
Coxibs	0.45 (0.45-0.46)	<0.001
Inhalable corticosteroids	0.45 (0.45-0.46)	<0.001
Ipratropium	0.45 (0.45-0.46)	<0.001
Selective beta-2 stimulants	0.45 (0.44-0.46)	<0.001
Antidepressants	0.46 (0.45-0.47)	<0.001
Benzodiazepines	0.46 (0.45-0.46)	<0.001
Benzodiazepine-related drugs	0.46 (0.45-0.47)	<0.001
Derivatives of benzodiazepines	0.46 (0.45-0.46)	<0.001
Hydroxyzine	0.46 (0.45-0.46)	<0.001
Migraine drugs	0.46 (0.45-0.47)	<0.001
Nitrofurantoin	0.46 (0.45-0.47)	<0.001
Paracetamol	0.46 (0.45-0.47)	<0.001
Propiomazine	0.46 (0.45-0.46)	<0.001
Trimethoprim	0.46 (0.45-0.47)	<0.001
Estrogens	0.47 (0.46-0.48)	<0.001
Gestagens	0.47 (0.46-0.48)	<0.001
Pivmecillinam	0.47 (0.46-0.48)	<0.001
Thyroid hormones	0.47 (0.46-0.48)	<0.001
Anti-conception drugs	0.65 (0.64-0.66)	<0.001

### List of prescription drugs excluded in Model 3

Antidepressants

Benzodiazepines

Benzodiazepine-related drugs

Derivates of benzodiazepines

Hydroxyzine

Migraine drugs

Nitrofurantoin

Paracetamol

Propiomazine

Trimethoprim

Estrogens

Gestagens

Pivmecillinam

Thyroid hormones

Anti-conception drugs

## Discussion

This study analysed morbidity-adjusted treatment with prescription drugs. We found that the gender difference in prescription drug use may to a large part be explained by gender-related morbidity, but there is still a gender difference at 18% that has not been explained. After adjustment for age and multi-morbidity, the odds of having prescription drugs for males, compared to females, was 0.45. After adjustment for age, multi-morbidity and prescription drugs used to treat diseases that afflict females to a higher extent than males, the odds ratio of having prescription drugs for males increased to 0.82.

Our study included the total population above 20 years of age in Östergötland, a county representative of the population in Sweden in terms of age [19].

The exclusion of anti-conception drugs had the strongest effect on the results, causing the OR to increase from 0.45 to 0.65. Since many healthy females use anti-conception drugs, these drugs should not be included in future studies comparing prescription drugs between males and females.

We expected a strong effect when excluding prescription drugs that are used to treat climacteric complaints, but this exclusion only increased the OR from 0.45 to 0.47. This could partly be explained by more restrictive prescription of hormone replacement therapy according to new guidelines [32].

Overall, with the exception of anti-conceptive drugs, the effects on the OR were small when analysing the exclusion of prescription drugs one by one, but the overall effect on the OR was substantial. Even though the effect of each individual prescription drug was low, the aggregative effect may be high if doctors commonly prescribe these drugs to females.

We expected that the exclusion of prescription drugs used to treat hormone-sensitive breast cancer would lead to overestimation of the effect of gender-related morbidity on the gender difference in drug prescription. However, we estimated that the effect of including these prescription drugs would be cancelled out by the effect of prescription drugs used to treat prostate cancer [18].

After the adjustments made in this study there was still a gender difference of 18% in prescription drug use, with a higher prescription rate among females. This may partly be explained by the fact that females have a higher doctor consultation rate compared to males [13-15]. It has also been shown that females tend to seek more preventive care compared to males, who seek emergency care at a higher rate, which may indicate that females seek health care earlier and have a higher likelihood of having prescription drugs [15,33]. It is also possible that the difference in prescription of drugs may be doctor-related, as has been observed in recent studies [34,35].

The gender-related morbidity described in the report from the Swedish National Institute of Public Health that was the basis for this study comports well with the gender-related morbidity seen in other studies [36-40]. In the present study, 66% of the study population used prescription drugs, in line with other studies [11,34,41].

This study shows that when comparing the prescription of drugs between males and females, it is very important to take into account gender-related morbidity. Still, after adjustment for multi-morbidity and gender-related morbidity, there remains a gender difference. Thus, other factors than pure medical ones seem to affect the prescription of drugs. Future research needs to focus on that.

### Limitations

Most of the diseases that females are afflicted with to a higher extent in the report from the Swedish National Institute of Public Health are diseases that may commonly be treated with only one prescription drug, i.e. hypothyroidism, cystitis and migraine. Even if we used Swedish guidelines when we identified the prescription drugs that are used to treat diseases that females are afflicted with to a higher extent [30,31] there is a risk that the doctors do not follow guidelines. This could lead to both under- and overestimation of the results when prescription drugs with an indication for treatment of diseases that females are afflicted with to a higher extent are used for other purposes and vice versa.

As mentioned before, because of the logistic model for example dementia was not included among the diseases that affect females to a higher extent than males and consequently the prescription drugs used to treat dementia were not excluded from the study. This may have led to lower ORs and underestimation of the effect of gender-related morbidity on the gender difference in drug prescription.

The chosen cut-off point for the number of DALYs (7,500) may have been set too high and the chosen cut-off point for gender difference in DALYs (20%) may have been set too low. This may have led to underestimation of the effect of gender-related morbidity on the gender difference in drug prescription.

The classification of anti-conception drugs as prescription drugs may differ between different countries. In Sweden they are classified as prescription drugs and are included in all prescription drug statistics. The results of the present study cannot be generalised to countries where anti-conception drugs are not classified as prescription drugs.

## Conclusion

Gender-related morbidity and the use of anti-conception drugs may explain a large part of the difference in prescription drug use between males and females but still there remains a difference between the genders at 18%. This implicates that it is of importance to take the gender-related morbidity into consideration, and to exclude anti-conception drugs, when performing studies regarding difference in drug use between the genders.

## Competing interests

The authors declare that they have no competing interests.

## Authors’ contributions

JS drafted the manuscript and participated in the design of the study. PM, LB and JST helped to draft the manuscript. AH performed the statistical analysis, helped to draft the manuscript, handled the data set and designed the study. All authors read and approved the final manuscript.

## Pre-publication history

The pre-publication history for this paper can be accessed here:

http://www.biomedcentral.com/1471-2458/14/329/prepub
